# In Vitro Studies of Fermented Korean Chung-Yang Hot Pepper Phenolics as Inhibitors of Key Enzymes Relevant to Hypertension and Diabetes

**DOI:** 10.3390/foods8100498

**Published:** 2019-10-14

**Authors:** Su-Jung Yeon, Ji-Han Kim, Won-Young Cho, Soo-Ki Kim, Han Geuk Seo, Chi-Ho Lee

**Affiliations:** 1 Department of Agricultural, Life and Environmental Science, Tottori University, Tottori 680-8550, Japan; sujung0811@gmail.com; 2 AgResearch (Grasslands Research Centre), Palmerston North 4442, New Zealand; aaddoo@nate.com; 3 Department of Food Science and Biotechnology of Animal Resources, Konkuk University, Seoul 05029, Korea; ready1838@naver.com (W.-Y.C.); hgseo@konkuk.ac.kr (H.G.S.); 4 Department of Animal Science and Technology, Konkuk University, Seoul 05029, Korea; sookikim@konkuk.ac.kr

**Keywords:** pepper, fermentation, hyperglycemia, angiotensin I-converting enzyme (ACE) inhibition, antioxidant

## Abstract

This study was investigated to evaluate the antioxidant activity, the angiotensin I-converting enzyme (ACE) inhibition effect, and the α-amylase and α-glucosidase inhibition activities of hot pepper water extracts both before and after their fermentation. The fermented pepper water extract (FP) showed significantly higher total phenol content, 1,1-diphenyl-2-picrylhydrazyl (DPPH) radical inhibition effect, metal chelating activity and ACE inhibition activity compared to the non-fermented raw pepper water extract (RP) (*p* < 0.05). Meanwhile, the FP showed lower α-amylase and higher α-glucosidase inhibitory activities, but the RP showed similar levels of α-amylase and α-glucosidase inhibitory activities. Taken together, these results suggested that fermented pepper extract using water should be expected to have potentially inhibitory effects against both hyperglycemia and hypertension.

## 1. Introduction

Hypertension is a condition of oxidative stress, therefore a decline in the antioxidant system can be fatal in hypertension patients, which can cause atherosclerosis and other hypertension-induced organ injuries [1]. Several researchers have reported the side effects of artificial antioxidants such as potential health hazards, for example, carcinogens [2,3]. Additionally, concerns about synthetic products have caused increasing interest in natural antioxidants made from food or other bioresources. Recently, there has been active interest in the research-based development of natural antioxidants such as vitamin E and ascorbic acid [3,4,5].

Meanwhile, angiotensin I is activated by renin secreted from the kidney; and angiotensin I-converting enzyme (ACE) changes angiotensin I to angiotensin II [6]. Angiotensin II leads to cardiovascular disease and vasoconstriction [7]. There are two kinds of prescription for treating hypertension—angiotensin converting enzyme inhibitor (ACEI) and angiotensin receptor blocker (ARB). Mostly, ACEI is used first with patients, because ACEI significantly reduces all-cause mortality at a rate greater than that of ARB [8,9]. Lee et al. [7] reported that general ACEI—such as captopril, quinapril, enalapril, lisinopril—has side effects such as cough, hypotension, and inflammatory response [10]. Therefore, the development of treatments having no side effect is needed, and much research along these lines has been reported [11,12].

α-amylase and α-glucosidase are related to the hydrolysis of dietary carbohydrates and the absorbance of glucose from the small intestine to the blood, respectively [13,14]. Control of these enzymes can play a key role in treating patients with diabetes mellitus [6,13]. Several inhibitors of these enzymes—such as acarbose, trestatin, and amylostatin—have recently shown adverse effects such as abdominal distention and meteorism [15]. This occurs because of the fermentation of undigested carbohydrates by anaerobic bacteria in the colon [13,16,17]. Thus, comparatively riskless inhibitors should be developed.

Pepper is currently being widely used for cooking in both traditionally and contemporary cuisine, and it contains a variety of phytochemicals such as capsaicin and ascorbate [18]. Capsaicin has an anti-obesity effect [19], can be used as an analgesic [20], and is an antioxidant [21]. However, due to its pungency, it is difficult to use it as a food additive in the food industry. In our previous studies, pepper fermented by *Bacillus licheniformis* showed significantly decreased capsaicin content, and pungency was decreased also, and this microorganism can utilize capsaicin as an energy source [22,23,24].

This study was performed to evaluate the antioxidant activity and the key enzyme inhibition effects related with hypertension and diabetes mellitus using fermented pepper, for the purpose of developing new functional food ingredients.

## 2. Materials and Methods

### 2.1. Materials

The pepper was obtained from a local market (Hwayang-dong, Seoul, Korea). All reagents were obtained from Sigma-Aldrich (St. Louis, MO, USA).

The pepper was blended and freeze dried. To prepare fermented pepper, 40 g of powdered pepper was mixed with 1 L of distilled water. After sterilization, the sample was inoculated with 5% of *Bacillus licheniformis* SK1230. It was then incubated for 11 days at 37 °C in a shaking incubator (Jisico, J-SIL-R, Seoul, Korea). After fermentation, the fermented pepper was freeze dried. For the raw pepper, the same amount of sterilized (inactive) Luria–Bertani (LB) broth (NaCl 10 g/L, tryptone 10 g/L, and yeast extract 5 g/L) was added (to substitute for the presence of microorganism), then freeze dried. Powdered raw pepper and fermented pepper were stored at −20 °C before use and were used as samples in this experiment. According to previously reported capsaicin analysis method [22], the amount of capsaicin in raw hot pepper was 1.31 mg/g and that of fermented pepper was significantly decreased to 1.20 mg/g (*p* < 0.05).

To prepare the ACE solution, 1 g of commercial rabbit lung acetone powder (Sigma-Aldrich, St. Louis, MO, USA) in 10 mL buffer (0.05 M Na_2_B_4_O_7_ : 0.2 M H_3_BO_3_ = 135 : 165, pH 8.3) was stirred at 4 °C for 24 h in dark room. Then, it was centrifuged at 12,000 rpm for 30 min. The supernatant was used as the ACE solution [25].

Dinitrosalicylic acid (DNS) reagent was prepared by mixing 1 g of DNS with 50 mL of distilled water. Then, 30 g of sodium potassium tartrate tetrahydrate was slowly added. 20 mL of 2 N NaOH was also added to the mixture, and distilled water was added until the mixture totaled 100 mL [26].

### 2.2. Extraction

The extract was prepared by means of a modified method published by Kwon et al. [13]. Of the sample 0.4 g was extracted with 10 mL of distilled water, over a period of 3 h. After centrifuging at 9300× *g* for 10 min, the supernatant was filtered using Whatman filter paper No. 2, and it was used for experimentation. (The “raw pepper water extract” and the “fermented pepper water extract” were expressed as RP and FP in this study, respectively.)

### 2.3. Total Phenol Compounds Content

The total content of the phenol compounds was determined according to literature [27]. Of the extract 30 μL was mixed with the same amount of 95% ethanol, 150 μL of distilled water, and 15 μL of 1 N Folin–Ciocalteu reagent. This mixture was allowed to react for 5 min, after which time 5% Na_2_CO_3_ 30 μL was added. After 1 h, absorbance was measured at 725 nm. Gallic acid was used to establish a standard curve, and results were expressed as mg of gallic acid equivalents per gram of dried sample weight.

### 2.4. 1,1-Diphenyl-2-Picrylhydrazyl (DPPH) Radical Scavenging Activity

DPPH (1,1-diphenyl-2-picrylhydrazyl) radical scavenging activity by Blois [28] were performed to evaluate antioxidant activity of the extract. Of the extract, 20 μL was reacted with 180 μL of 0.1 mM DPPH in a 96 well plate. The absorbance was recorded at 517 nm after 30 min in a dark room. Ethanol, instead of extract, was used for the control. DPPH radical inhibition was calculated by:

Inhibition (%) = (1 − A_517_(extract)/A_517_(control)) × 100 (1)

### 2.5. Metal Chelating Activity

Another antioxidant activity of extract was performed by metal chelating activity [29]. Of the extract, 100 μL was mixed with 50 μL of 2 mM ferrous chloride, and the reaction was started by adding 0.2 mL of 5 mM ferrozine. Then, absorbance was recorded at 562 nm after 10 min in a dark room. Distilled water, instead of extract, was used for the control. Metal chelating activity was calculated by:

Inhibition (%) = (1 − A_562_(extract)/A_562_(control)) × 100 (2)

### 2.6. Angiotensin I-Converting Enzyme (ACE) Inhibition Effect

The ACE inhibition effect was performed by Chushman et al.’s method [25]. An extract of 40 μL, 12.5 mM of hippuryl-histidyl-leucine in an 80 μL buffer (0.05 M Na_2_B_4_O_7_ : 0.2 M H_3_BO_3_ = 135 : 165, 0.4 M NaCl, pH 8.3) and an ACE solution of 80 μL was reacted for 30 min in a 37 °C water bath. Then, 200 μL 1 N HCl was added to stop the reaction. Ethylacetate (1.2 mL) was added, and the mixture was vortexed for 5 min. After centrifugation at 3000 rpm for 15 min, the supernatant of 0.8 mL was allowed to stand at 90 °C for removal of ethylacetate. After that, the residue was mixed with 2.4 mL of distilled water, and the absorbance was recorded at 228 nm (OPTIZEN 2120UV, Mecasys Co. Ltd., Daejeon, Korea). The blank contained HCl from the beginning to prevent the reaction of substrate and enzyme. Distilled water, instead of extract, was used for the control. The ACE inhibition activity was calculated as below:

Inhibition (%) = (1 − (A_228_(extract) − A_228_(extract blank))/(A_228_(control) − A_228_(control blank))) × 100 (3)

### 2.7. α-Amylase Inhibition Effect

The α-amylase inhibition effect was evaluated by a modification of the assay described by the Worthington Enzyme Manual [26]. Of the extract, 30 μL and 0.02 M of sodium phosphate buffer (pH 6.9) containing 400 U/mL of α-amylase was mixed and pre-incubated at 25 °C for 10 min. After adding 30 μL of 1% starch solution in 0.02 M sodium phosphate buffer (pH 6.9) to the mixture, it was stored at 25 °C for 10 min. To stop the reaction, 60 μL of DNS color reagent was added. Then, it was allowed to stand in a boiling water bath for 5 min, prior to cooling to room temperature. After dilution with distilled water in the amount of 0.4 mL, absorbance was read at 540 nm. Only 0.02 M sodium phosphate buffer (pH 6.9), instead of buffer containing α-amylase and extract, was used for the blank and the control, respectively. Percentage of inhibition was calculated as follows:

Inhibition (%) = (1 − (A_540_(extract) − A_540_(blank))/A_540_(control)) × 100 (4)

### 2.8. α-Glucosidase Inhibitory Activity

The α-glucosidase inhibitory activity was measured by the modified method of Ranilla et al. [6]. Of the extract, 50 μL and 0.1 M potassium phosphate buffer (pH 6.9) containing 1 U/mL of α-glucosidase, was mixed and pre-incubated at 25 °C for 10 min. After adding 50 μL of 5 mM p-nitro-phenyl-α-D-glucopyranoside in a 0.1 M potassium phosphate buffer (pH 6.9) to the mixture, absorbance was measured at 405 nm. Then, after incubating the mixture at 25 °C for 5 min, absorbance was read at 405 nm. Potassium phosphate buffer (0.1 M, pH 6.9), in the place of extract, was used for control. Inhibitory activity was calculated as below:

Inhibition (%) = (1 − (ΔA_405_(extract))/ΔA_405_(control)) × 100 (5)

### 2.9. Statistical Analysis

All results were stated as mean ± standard error of mean (SEM) and, analyzed by *t*-test using SPSS 18.0 (SPSS Inc., Chicago, IL, USA). The experiments were performed in triplicates. Significant difference was evaluated by *p* < 0.05.

## 3. Results

### 3.1. Amount of Total Phenol Contents

Total phenol contents in extracts from raw and fermented peppers varied from 150 to 250 μg/mL (Table 1), and it was similar to the range reported by Kwon et al. [18]. The total phenol content of FP was 40% higher than that of the raw pepper extract significantly (*p* < 0.05).

### 3.2. Antioxidant Activity

Antioxidant activities were determined by both DPPH radical inhibition assay and metal chelating assay (Table 2). The FP showed significantly higher radical inhibition activity and chelating activity than did those of the RP (*p* < 0.05). Meanwhile, metal chelating activity was significantly higher than DPPH radical inhibition activity in both RP and FP (*p* < 0.05).

### 3.3. ACE Inhibition Effect

The inhibition rate of angiotensin I-converting enzyme is exhibited in Table 3. The water extract of fermented pepper showed a significantly higher inhibition rate as compared to that of raw pepper extract (*p* < 0.05).

### 3.4. α-Amylase Inhibition and α-Glucosidase Inhibitory Activity

α-amylase and α-glucosidase inhibition activity were seen in the RP and FP (Table 4). Fermented pepper extract exhibited significantly lower α-amylase and α-glucosidase inhibition rates than the RP (*p* < 0.05). Meanwhile, α-amylase and α-glucosidase inhibition activity in RP were similar each other (*p* > 0.05), but in FP, low α-amylase and high α-glucosidase inhibition activity were shown significantly (*p* < 0.05).

## 4. Discussion

Consistent with this study, Sim and Han [30] reported that the amount of phenolic compounds derived from red pepper seed on Kimchi significantly increased during fermentation (*p* < 0.05). Generally, phenolics exist as conjugated forms together with sugars or other moieties. Glucosidase is related to the hydrolysis of glycosidic linkages, and it leads to the release of free phenolics [31]. In this study, glucosidase inhibition activity of FP decreased significantly as compared to that of the RP (Table 4, *p* < 0.05). This means that the FP had higher glucosidase activity than the RP. Therefore, it suggested that it resulted in the release of free phenolics with increasing glucosidase activity during fermentation of the pepper.

Meanwhile, antioxidant activity generally correlates with the amount of phenolic compounds. Vattem et al. [31] reported that total phenolics and antioxidant activity correlated positively with glucosidase activity. Accordingly, significantly higher DPPH radical inhibition effect in the FP was suggested due to a higher total phenolics level and a lower glucosidase inhibition rate in comparison to the RP (Table 1 and Table 4, *p* < 0.05). There were several ways to protect oxidation. Elimination of over produced free radicals which can react with biomolecules and cause chronic diseases [32] and heavy metals by chelating agent from fermented pepper extract would be the way for antioxidation.

There have been several reports about ACE inhibition activities by fermented milk peptides [33], flavanol-rich foods [34], and various fermented foods such as soy sauce, fish sauce, natto, and cheese [35]. Actis-Goretta et al. [34] referenced that the ACE inhibition effect was related to amounts of phenolics and flavanols. Meanwhile, Okamoto et al. [35] implied, on the basis of their research, that an ACE inhibitor would be produced during fermentation. For example, cottage cheese—which has no maturing step in the manufacturing process—did not show ACE inhibition activity. By contrast, red cheddar, blue, and camembert cheese—all of which have a maturing process—showed ACE inhibition effect. In this study, pepper exhibited a significantly higher amount of total phenolics after fermentation (*p* < 0.05). A relationship with higher ACE inhibition activity than before fermentation has been suggested. Higher ACE inhibition means lower conversion of angiotensin I to angiotensin II, a potent vasoconstrictor, and it seems to be a strategic treatment to protect against hypertension—which is a representative complication of diabetes [6].

Ranilla et al. [6] recently reported that high phenolic-linked plants showed a high α-glucosidase inhibition rate with a low activity of α-amylase. Consistent with this result, FP—having a higher total phenol contents than the RP—had higher α-glucosidase inhibition activity with lower α-amylase inhibition significantly (*p* < 0.05). Martin and Montgomery [36] referenced a finding that such a result could have functionality regarding the potential controlling of glucose absorption and of adverse effects from high α-amylase inhibition activity.

**Figure 1:**
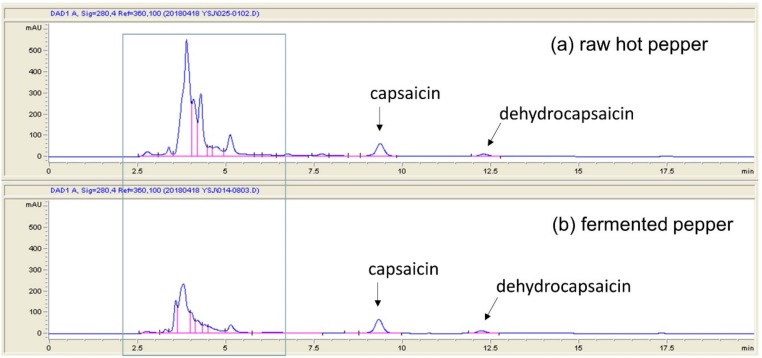
The chromatogram of raw hot pepper and fermented pepper.

In this study, we fermented pepper for 11 days with the intention of “capsaicin decrease” not “no capsaicin” in pepper for adjustable pungency. If the effect of fermented pepper is higher than that of raw hot pepper, it is thought to be resulted from the degradation of the ingredient of capsaicin or other fermentation by-product even if capsaicin remains in the fermented pepper. Indeed, the chromatogram of fermented pepper showed a greater area in some peaks compared to that of raw hot pepper (blue square in Figure 1) and what this compound is and whether it has functionality will be studied in future.

## 5. Conclusions

Fermented pepper extract using water was evaluated about antioxidant activity, ACE and enzymes relevant for hyperglycemia inhibition effects. Results showed significantly higher total phenol contents, DPPH radical inhibition activity, metal chelating activity, and ACE inhibition effect than those of RP (*p* < 0.05). Furthermore, lower α-amylase and higher α-glucosidase inhibition effects in FP could have inhibitory potential in diabetes. Therefore, it is suggested that water extract of fermented pepper might be appropriate for a functional prevention ingredient of hypertension and hyperglycemia as a natural resource.

## Figures and Tables

**Table 1:** The amounts of total phenol compound in pepper extract, both before and after fermentation (gallic acid μg/mL).

	RP	FP
Total phenol compound (gallic acid μg/mL)	156.47 ± 2.12 ^b^	224.55 ± 2.51 ^a^

RP, raw pepper extract prepared using water. FP, fermented pepper extract prepared using water. ^a,b^ Superscripts with different letters indicate significant difference (*p* < 0.05).

**Table 2:** Antioxidant activities of raw and fermented pepper extracts.

	RP	FP
DPPH radical inhibition (%)	52.00 ± 0.28 ^b B^	66.12 ± 0.72 ^a B^
Metal chelating activity (%)	67.32 ± 0.75 ^b A^	71.99 ± 0.82 ^a A^

RP, raw pepper extract prepared using water. FP, fermented pepper extract prepared using water. DPPH: 1,1-diphenyl-2-picrylhydrazyl. ^a,b^ Superscripts with different letters indicate significant difference between RP and FP (*p* < 0.05). ^A,B^ Superscripts with different letters indicate significant difference between DPPH radical inhibition and metal chelating activity (*p* < 0.05).

**Table 3:** Angiotensin I-converting enzyme (ACE) inhibition effects of raw and fermented water-prepared pepper extracts.

	RP	FP
ACE inhibition (%)	54.29 ± 0.71 ^b^	71.04 ± 0.58 ^a^

RP, raw pepper extract prepared using water. FP, fermented pepper extract prepared using water. ACE: angiotensin I-converting enzyme. ^a,b^ Superscripts with different letters indicate significant difference (*p* < 0.05).

**Table 4:** Inhibition rates of α-amylase in raw pepper water extract (RP) and fermented pepper water extract (FP).

	RP	FP
α-amylase inhibition (%)	25.15 ± 0.27 ^a NS^	8.26 ± 0.16 ^b B^
α-glucosidase inhibition (%)	24.32 ± 0.86 ^a^	14.47 ± 0.67 ^b A^

RP, raw pepper extract prepared using water. FP, fermented pepper extract prepared using water. ^a,b^ Superscripts with different letters indicate significant difference between RP and FP (*p* < 0.05). ^A,B^ Superscripts with different letters indicate significant difference between amylase and glucosidase inhibition (*p* < 0.05). ^NS^ Not significant between amylase and glucosidase inhibition (*p* > 0.05).
